# Supplementary material for: The influence of metformin transporter gene SLC22A1 and SLC47A1 variants on steady-state pharmacokinetics and glycemic response
Source: PLoS One. 2022 Jul 29;17(7):e0271410. doi: 10.1371/journal.pone.0271410 (PMC9337647; doi:10.1371/journal.pone.0271410)
Supplement: Attachment — Submitted filename: Response to Reviewers.docx [file pone.0271410.s001.docx]

Vitarani D.A Ningrum

Department of Pharmacy

Universitas Islam Indonesia

Yogyakarta, Indonesia

June 29, 2022

**Academic Editor**

**PLOS ONE**

Thank you for your helpful comments. We have revised our paper accordingly and feel that your comments helped clarify and improve our paper. Please find below our responses:

1. **Comments From Editor:**

Please provide additional details regarding participant consent. In the Methods section, please ensure that you have specified (1) whether consent was informed and (2) what type you obtained (for instance, written or verbal). If your study included minors, state whether you obtained consent from parents or guardians. If the need for consent was waived by the ethics committee, please include this information

**These suggested edits have been completed (lines 108 – 117)**

2. **Comments From Editor:**

The presentation can be improved; at some places, English grammar/usage can be improvised

**We have rechecked the grammatical accuracy of the manuscript**

3. **Comments From Editor:**

In table 2, comma instead of period is used as the decimal point indicator at some places.

**These suggested edits have been completed (Table 2)**

4. **Comments From Editor:**

% values are actually not noted in table 2

**We have added % values in Table 2.**

5. **Comments From Editor:**

For all tables & figures, provide keys for the abbreviations/acronyms that are used

**These suggested edits have been added (Table 1., Table 2., and Table 3.)**

6. **Comments From Reviewer:**

At some places authors mentioned they have recruited T2DM patients taking metformin for eight weeks.
But some places e.g. in methodology in Measurement of the Glycemic Response: its mentioned The FBG and GA of T2DM patients given metformin monotherapy were measured before and after the continuous administration of metformin 500 mg twice daily for six weeks.
Please explain

**One of the inclusion criteria for this study is that the subject had been using metformin for a minimum of two weeks to ensure that the steady-state concentrations were achieved. A glycemic control examination was then performed at the beginning and six weeks after the routine use (the patient was confirmed to be compliant with the use of metformin during these six weeks). Therefore, in line with the reviewer's input, the author has revised the results and discussion section by adding such information (lines 106-107, 303-304)**

7. **Comments From Reviewer:**

Minor concerns
Please give full form of MALA, eGFR.

**These suggested edits have been completed (eGFR line 40; MALA line 228)**

8. **Comments From Reviewer:**

Spelling error:
In introduction “Hipoglycemic”.

**We have revised the word to hypoglycemia (line 61)**

We appreciate your careful evaluation of our work and hope that this revision has addressed the comments and meets with your approval. We await your response.

Yours Sincerely,

Vitarani D.A Ningrum
